# Provider experiences of virtual reality in clinical treatment

**DOI:** 10.1371/journal.pone.0259364

**Published:** 2021-10-29

**Authors:** Christine Vincent, Margaret Eberts, Tejal Naik, Victoria Gulick, C. Virginia O’Hayer

**Affiliations:** 1 Sidney Kimmel Medical College at Thomas Jefferson University, Philadelphia, PA, United States of America; 2 Department of Medicine at Thomas Jefferson University, Philadelphia, PA, United States of America; 3 Information Services & Technologies at Jefferson Health, Philadelphia, PA, United States of America; 4 Department of Psychiatry & Human Behavior at Thomas Jefferson University, Philadelphia, PA, United States of America; Prince Sattam Bin Abdulaziz University, College of Applied Medical Sciences, SAUDI ARABIA

## Abstract

**Background:**

Virtual reality (VR) has proven effective in the treatment of specific phobias and trauma particularly when in-vivo exposure therapy might be costly (e.g. fear of flying, combat scenes). Similarly, VR has been associated with improvement of chronic pain and of acute pain during medical procedures. Despite its effectiveness as a healthcare tool, VR technology is not well-integrated into common practice. This qualitative study aims to explore the provider perception of the value of VR and identify barriers to VR implementation among healthcare providers.

**Methods:**

A 66-item self-report survey was created to examine application of VR to clinical practice, perceived value of this treatment, ease of learning the technology, billing considerations, and other obstacles. 128 providers (MDs and PhDs) who were located in the United States and had used VR as a therapeutic tool in the past year were identified through research papers, as well as user lists and news articles from VR application websites. Of the 128 providers contacted, 17% (22) completed our online self-report measure. Of these, 13% of respondents (N = 17) completed greater than 75% of the questionnaire and were considered completers. Provider responses were collected over a one-month period and qualitatively analyzed.

**Results:**

The majority of providers were from an academic institution (n = 12, 70.6%), and all providers practiced in the outpatient setting. Providers most commonly reported using VR for the treatment of acute pain and/or anxiety related to medical procedures (n = 11, 64.7%), followed by specific phobia (n = 6, 35.3%) and social phobia (n = 6, 35.3%). All providers agreed VR is a valuable tool they would recommend to colleagues. The majority (n = 15, 93.8%) believed VR helped their patients progress in treatment, compared with other methods. Providers cited the ability to individualize treatment (n = 14, 87.5%) and increase patient engagement (n = 15, 93.8%) as main benefits of VR. A minority reported negative feedback from patients about content (n = 4, 25%) or about the technology in general (n = 6, 37.5%), whereas all reported some form of positive feedback. The slight majority (n = 10, 58.8%) of providers did not find transitioning to VR difficult. Of those who did, cost was the most commonly cited barrier (n = 6). Regarding reimbursement, only 17.6% (n = 3) of providers reported the ability to bill for VR sessions. Most providers (n = 15, 88.2%) received training on their VR platform which they found beneficial. Comparing the trained and untrained groups found no significant difference in VR comfort level (p = 0.5058), the value of VR in practice (p = 0.551) or whether providers would recommend VR to others (p = 0.551), though sample sizes were small.

**Conclusions:**

In corroboration with previous research, this study demonstrates that VR is well-received by patients and providers, allowing increased patient engagement and treatment individualization. However, associated costs, including an inability to bill for this service, can present a barrier to further implementation. These findings will guide further development of virtual reality as a standardized tool in psychiatry and pain management.

## 1 Introduction

Virtual reality (VR) is an innovative technology that allows the user to vividly experience scenarios via immersive, multi-sensory information, typically delivered via headset including visual and auditory cues. Clinical applications of this technology include the treatment of specific phobias, trauma, eating disorders, and also management of acute and of chronic pain [1, 2]. Recent years have seen VR applications developed and integrated into many realms of psychiatry and behavioral medicine, particularly for the treatment of anxiety disorders [3–6], post-traumatic stress disorder (PTSD) [7], and pain management [8, 9]. Randomized controlled trials have found VR therapy to be as effective for anxiety disorder treatment as the current gold standards, in vivo exposure and cognitive behavioral therapy [9–12]. Advantages of Virtual Reality Exposure Therapy (VRET) over in vivo exposure for the treatment of phobias and other anxiety disorders include high levels of control, safety of treatment, and maximization of the fit between the patient’s feared stimuli and the exposure [4, 11, 13]. This advantage is most striking in situations in which exposure therapy may not be feasible including fear of flying or fear of small spaces [4]. Among patients diagnosed with PTSD, especially those from active service/combat, use of VRET has been found to significantly reduce both symptom severity as well as the number of individuals who met diagnostic criteria for PTSD [7, 14].

Immersive VR experiences have shown reduction in subjective pain scores when used as a distraction technique for both chronic pain secondary to underlying illness (eg. fibromyalgia, lower back pain, diabetic neuropathy, and chemotherapy associated pain) [9] as well as acute pain during medical procedures (eg. postoperative wound dressing changes, labor analgesia, wound dressing changes) [3, 15–18]. Examples of immersive VR experiences include “SnowWorld,” an interactive game developed for pediatric burn victims [17], enchanted forests and dream castle scenery for relaxation, and guided hypnosis sessions [9].

Providers and patients who have used VR have overwhelmingly positive experiences to share [1, 19, 20]. Health professionals currently using VR in practice highlight benefits including flexibility, adaptability, and accessibility of the technology. Patients endorse VR to be a beneficial adjunct or stand-alone therapy, especially to manage their chronic cancer pain [1], or anxiety related to critical medical conditions [20, 21]. Interestingly, health professionals *not* currently using VR share the perceived therapeutic benefits identified by their colleagues regarding VR [22, 23]. However, despite the known benefits of VR, many providers lack knowledge and resources regarding how to feasibly integrate VR into their practices [24]. Surveys of providers not currently using VR suggest reluctance may stem from beliefs about required training, financial costs, uncertainty of benefits, possible negative reactions from patients, and whether use may compromise the physician-patient relationship [19, 24, 25]. The lack of more widespread integration of VR into clinical practice suggests the need for further research into whether these anticipated barriers were encountered among providers who integrated VR into their practice.

The aim of the present study is to explore how psychiatric and healthcare providers are using VR, how useful they find this tool, and any barriers to implementation. In light of research demonstrating the effectiveness of VR use in the treatment of psychiatric disorders, and pain management, our hope is to synthesize the experiences of providers using VR, including debunking any myths that may be preventing other providers from adopting this therapeutic tool.

Our objective is to better understand the implementation process and barriers experienced by those using VR in mental health and pain management. Based on the current literature, there is sufficient provider and patient interest in incorporating VR into their treatment plans, but there is also a feasibility roadblock—many providers believe they do not have the knowledge or time to invest in the novel VR technology [25]. By gathering information from current providers on the value VR has for their practice, the process of onboarding, and the technicalities of use, we aim to understand the achievability of disseminating VR as a more standardized treatment in psychiatric practices.

## 2 Methods

### 2.1 Study design

Given the paucity of information regarding the experience of providers in implementing VR, we designed a cross-sectional, qualitative study, in efforts to capture a wide variety of provider experiences. Study procedures were approved by the Thomas Jefferson University Institutional Review Board (IRB #20E.634) in June 2020. Eligible participants were contacted via email and were informed about the study. No compensation was provided for participation in the study. No known database of this population exists, so potential participants were selected from a random sampling of research papers, user lists, and news articles from VR application websites.

### 2.2 Participants

The authors conducted an extensive review of VR providers, then contacted those for whom contact information was available to invite them and/or their colleagues to participate in this survey. Prospective providers were identified through published literature regarding VR, news articles from VR application websites, and through their affiliation with various VR platforms and centers including AppliedVR, Brain Power, BraveMind, HealthTech, KindVR, Limbix, Oncomfort, Psious, SnowWorld, Verapy, Virtual Reality Medical Center, and Virtually Better. Inclusion criteria were limited to physicians and PhD-level providers, based in the United States, who had used VR in their clinical practice in the past year. Exclusion criteria was limited to providers who had not used VR in their clinical practice in the past year, or who were practicing outside of the United States. Participants were emailed the questionnaire on June 18^th^ 2020. Out of 128 health care providers (HCPs) who were contacted, 22 HCPs responded to the survey (17%). 18 of these 22 had complete datasets (defined as >75% of survey completed) and 4 of these 22 were excluded due to incomplete data (4%, 4%, 18%, and 75% of survey completed). Of these 18 complete datasets, one more was excluded as the participant only used VR as a calming technique for office staff and not for use with clients/patients. Thus, 17 complete datasets were used in the final data analysis. Out of these 17 participants, the majority work in an academic institution and use VR with outpatient populations. The majority have been using VR for over 3 years in their clinical practice and spend less than 10% of their clinical time using VR.

### 2.3 Measures

This study used an online, self-administered questionnaire built in the Qualtrics survey platform. Given that no survey of its kind has been published, this questionnaire was created by the authors. As such, measures of reliability and validity are not known for this survey. The 62 item self-report instrument includes five main sections. Section one is ‘General VR use’ (6 questions). Questions include their primary care setting, which VR platforms are used in their practice, and for how long they had been using VR in their practice. Section two is ‘Perceptions of the value of VR in clinical practice’ (17 questions). Questions include a scale from strongly agree to strongly disagree where participants responded to if VR has been a valuable tool to them, if it helps them to more accurately diagnose their clients/patients, and if they have received positive feedback from their clients/patients regarding the use of VR. Section three is ‘Process of onboarding VR in a clinical practice’ (23 questions). Questions include how they heard about VR as an option for their practice, if they found it a challenge to transition to using VR, and how they addressed each challenge they identified. Section four is ‘Billing and reimbursement of VR in clinical practice’ (3 questions). Questions include if they are able to bill the client/patient’s insurance for the session and whether they are able to use an enhanced billing rate. Section five is ‘Clinical use of VR in the provider’s practice’ (13 questions). Questions include which client/patient diagnoses they are currently using VR with, what percentage of these specialized populations they are using VR with, and in which location they engage in VR treatment. The questionnaire consisted of matrix tables, multiple choice questions, check-all-that-apply questions, free response questions, and yes/no questions. See appendix for a complete list of questionnaire items.

### 2.4 Study procedures

An online, self-administered questionnaire designed to measure provider perceptions of their usage of VR within their practice was distributed to selected providers via email. If no response occurred following two weeks, a follow up email was sent. If no response ensued after another two weeks, the provider was considered a non-responder. Providers were also asked to forward the survey to any colleagues using VR in their clinical practice. Responses were all anonymous.

Survey data were collected between June 2020 and July 2020. Participants completed the questionnaire in an average of 8 minutes (range: 4 mins to 14 mins). All collected data were maintained on Qualtrics, an approved HIPAA-compliant survey software. No physical copies were maintained. No Protected Health Information (PHI) was collected, and emails/names were not associated with provider responses. Response frequencies (number of respondents and percentage of respondents) were calculated for each item.

## 3 Results

### 3.1 Respondent characteristics and VR usage

**Table 1** reports the breakdown of the relevant demographic information for all respondents included in the study. Between June and July 2020, 128 healthcare providers meeting the inclusion criteria were approached via email to participate. Twenty-two providers responded, 17 of which completed more than 75% of the survey and have been included in the results. Of the remaining five providers, one exclusively used VR for staff (not with patients/clients) and four completed 75% or less of the survey. Of note, questions were not mandatory and providers were permitted to skip survey questions. Further, some questions permitted respondents to select multiple answers. As a result, the total number of respondents for each question may vary. Percentages were calculated using the number of respondents for each question (N), which varies by question.

**Table 1:** VR in practice: Location, population, and time using VR.

	N (%) *
PROVIDER CLINICAL SETTING	
Academic institution	12 (70.6)
Community practice (Individual)	3 (17.6)
Other- Outpatient specialty medical clinic	1 (5.9)
Other- Clinical VR programmer/designer/researcher	1 (5.9)
PROVIDER CLINICAL POPULATION	
Mix of outpatient and inpatient	8 (47.1)
Outpatient only	9 (52.9)
LOCATION OF PATIENT VR USAGE	**
In person (outpatient)	14 (82.4)
In person (inpatient)	8 (47.1)
Client/patient independent use (client/patient uses app on their own)	6 (35.3)
Virtually (telehealth)	4 (23.5)
LENGTH OF TIME VR USED IN PRACTICE	
Less than 6 months	1 (5.9)
1–3 years	6 (35.3)
Over 3 years	10 (58.8)
PERCENT CLINICAL TIME SPENT USING VR	
0–10%	14 (82.4)
11–25%	2 (11.8)
26–50%	1 (5.9)
51–75%	0 (0)
76–100%	0 (0)

* A total of 17 provider responses are included in the study.

** Providers were permitted to select more than one response; thus percentages are calculated with N = 17, but do not add up to 100%.

**Fig 1:**
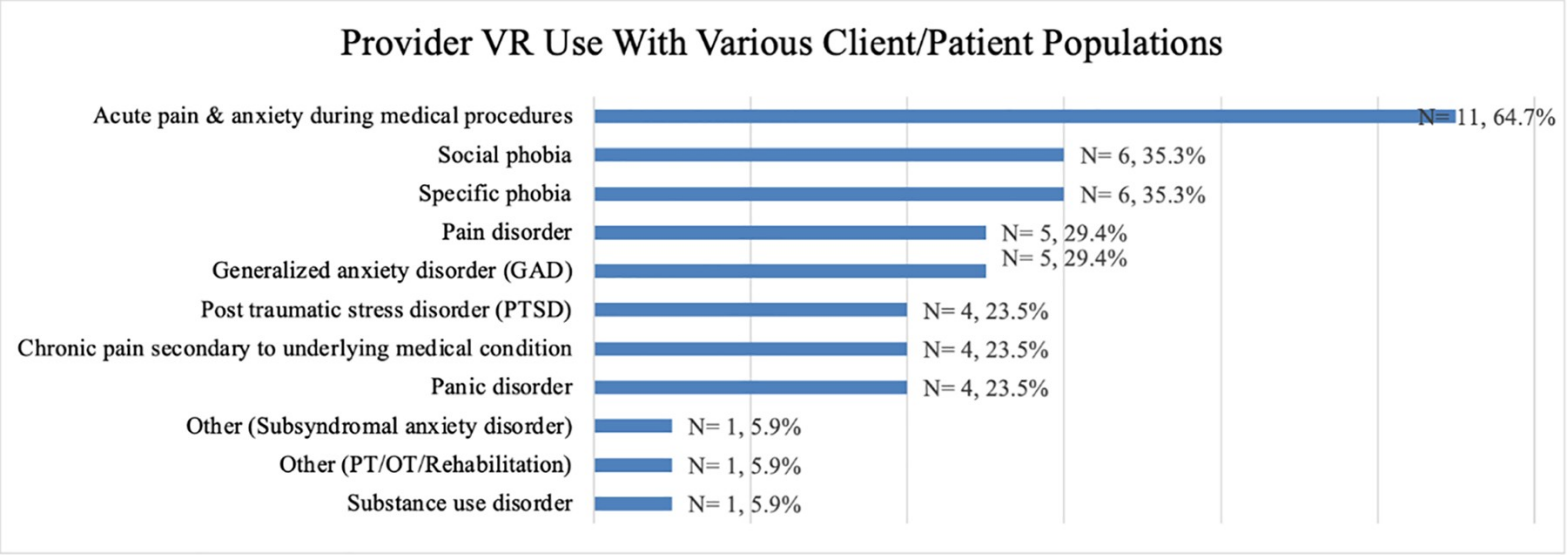
Provider VR use by percentage with various client/patient populations.

**Fig 1** reports the clinical conditions respondents report currently using VR to treat in practice. Many providers reported using VR to treat more than one patient population, thus are included in multiple columns. The most common patient population reported by providers was acute pain or anxiety during medical procedures (n = 11, 64.7%), followed by specific phobia (n = 6, 35.3%) and social phobia (n = 6, 35.3%). Some participants reported that they were not currently using VR, but in the future would like to use VR with various other client/patient populations: four providers reported wanting to use VR for chronic pain secondary to an underlying medical condition; another four said that they would like to use VR for acute pain/anxiety during medical procedures. Additionally, individual providers specified they would be interested in expanding VR use for each of the following patient populations: pain disorder, panic disorder, generalized anxiety disorder, substance use disorder, physical therapy/occupational therapy/Rehabilitation, and Other-not specified.

### 3.2 Provider perceptions of VR value in clinical practice

Respondents reported their degree of agreement with a variety of statements related to the value VR brings to their clinical practice. These data are summarized in **Table 2**. Interestingly, all providers agreed that VR treatment is a valuable tool in their practice (n = 16, 100%); with 75% (n = 12) of respondents strongly agreeing with this statement and 25% (n = 4) mildly agreeing. Additionally, all providers would strongly recommend the use of VR as a treatment tool to other providers in their field (n = 16, 100%). The vast majority of providers (n = 14, 87.5%) agree that VR allows for individualized treatment to patients and clients, and 93.75% (n = 15) of providers perceive their patients as more engaged in treatment involving VR compared to traditional methods. Additionally, 62.5% (n = 10) of providers reported that using VR has allowed them to have closer relationships with patients; of these providers, the most commonly cited therapeutic uses for VR included specific phobia (n = 5), social phobia (n = 5), acute pain/anxiety during medical procedures (n = 5), panic disorder (n = 3), and pain disorder (n = 3).

**Table 2:** Provider perceptions of VR value in clinical practice.

	Agree	Disagree
	N (%)	N (%)
VR treatment has been a valuable tool in my practice.	16 (100.0)	0 (0.0)
VR allows me to more accurately diagnose clients/patients.	6 (37.5)	10 (62.5)
VR allows me to individualize treatment to various clients/patients.	14 (87.5)	2 (12.5)
My clients/patients seem more engaged in their VR treatment than in traditional methods.	15 (93.8)	1 (6.3)
VR has helped my clients/patients with their progress in treatment compared with similar methods without VR.	15 (93.8)	1 (6.3)
I have received positive feedback from my clients/patients regarding the use of VR.	16 (100.0)	0 (0.0)
I have received negative feedback from my clients/patients regarding VR and…		
… they have attributed this to a problem with content (unrealistic scenes, grainy display, etc).	4 (25.0)	12 (75.0)
… they have attributed this to a problem with technology (cybersickness, applications are not intuitive to use, discomfort of equipment, etc).	6 (37.5)	10 (62.5)
… they have a problem with the general perception of VR as a treatment tool.	0 (0.0)	16 (100.0)
Using VR has allowed me to have closer relationships to my clients/patients.	10 (62.5)	6 (37.5)
When using VR, I spend more time with clients/patients than I would using other treatment techniques.	2 (12.5)	14 (87.5)
I often felt rushed when using VR with my clients/patients.	2 (12.5)	14 (87.5)
Using VR in my practice presents more technical issues than I would otherwise experience.	11 (68.8)	5 (31.3)
VR apps for therapeutic use are generally intuitive for me to use.	14 (87.5)	2 (12.5)
VR apps for therapeutic use are generally intuitive for my clients/patients to use.	12 (75.0)	4 (25.0)
I would recommend the use of VR as a treatment tool to other providers in my field.	16 (100.0)	0 (0.0)

All providers reported having received positive feedback from patients regarding the use of VR (n = 16, 100%). Two providers commented on the effectiveness of VR as a pain/distraction tool, especially with pediatric patients. One provider specifically noted, “VR has been very helpful as a distraction tool for pediatric oncology patients that are undergoing repeated trauma such as mediport accessing or anesthesia.” Similarly, the other pediatric provider commented, “We tend to use [VR] as a ‘set it and let the patient enjoy’ tool rather than staying with the patient. There is a little coaching at first to help them use it but after that, kids are pretty tech savvy and move through it easily.” Another provider who treats phobias commented that VR is “more feasible and cheaper for some applications such as airplanes.” Notably, one additional provider commented on the benefit of using VR as effective tele-rehab during the COVID-19 pandemic when in-person visits were limited.

In terms of use of VR for diagnostic purposes, 62.5% (n = 10) of providers did not agree that VR allowed them to more accurately diagnose patients. These providers most commonly used VR to treat patients with chronic pain or with acute pain/anxiety during medical procedures. Among the respondents who did endorse that VR allowed them to diagnose patients more accurately (n = 6, 37.5%), VR was used for conditions including specific phobia, social phobia, generalized anxiety disorder, PTSD, panic disorder, pain disorder, and substance use disorder.

While the majority of respondents endorsed that VR applications for therapeutic use are generally intuitive to use (n = 14, 87.5%), most respondents (n = 11, 68.8%) also endorsed that using VR in practice has presented more technical issues than traditional treatment. Interestingly, despite this, no respondents reported having received negative feedback from patients related to general perception of VR as a treatment tool.

Respondents did endorse having received some negative feedback from patients who received VR treatment in their clinical practice. Regarding negative feedback from patients, 25.0% (n = 4) of respondents reported have received negative feedback relating to VR content (e.g. unrealistic scenes, grainy display). This feedback was reported by providers who report using VR for specific phobia (n = 3), social phobia (n = 3), and panic disorder (n = 2).

Additionally, 37.5% (n = 6) of respondents endorsed having received negative feedback from patients relating to problems with the technology (e.g. cybersickness, discomfort of equipment). Providers who received this feedback reported using VR to treat specific phobia (n = 3), social phobia (n = 3), acute pain/anxiety (n = 3), chronic pain (n = 2), generalized anxiety disorder (n = 2), panic disorder (n = 2), pain disorder (n = 2) and PTSD (n = 1). One provider commented that they have received negative feedback related to patient boredom as their application has limited virtual environments.

### 3.3 Onboarding process

Providers indicated that they had heard of VR as an option through a variety of different avenues, the most common of which being self-directed research of available literature (n = 6, 35.3%). Other means of learning about VR included medical conferences (n = 3, 17.7%), websites (n = 2, 11.8%), colleagues in the provider’s same practice (n = 2, 11.8%), colleagues in a different practice (n = 2, 11.8%), the providers themselves are an inventor/pioneer of VR (n = 2, 11.8%), the provider’s training program (n = 1, 5.9%) and via working directly with VR applications previously (n = 1, 5.9%). The overwhelming majority of survey respondents reported their colleagues as being open to using VR as an option for therapy (n = 16, 94.1%). The single respondent who disagreed with this statement reported that their colleagues felt as though too much time and effort was required to learn how to use VR. They also cited fear that patients would not like VR as a form of therapy as the reason for their colleagues not being open to using VR.

Less than half of the providers, 41.2% (n = 7), found the transition to using VR in their practice challenging. Common reasons providers cited as to why the transition was challenging included that initial cost was a problem (n = 6), the technology was not intuitive to use (n = 4), training was required (n = 4), and ongoing technical support was not sufficient (n = 4). Additionally, two providers identified the lack of clinical staffing to administer the VR tool as the main barrier to more widespread adoption of VR in their clinical setting.

Reasons that the other 58.8% (n = 10) providers cited as to why their transition to VR use was not challenging included: feeling that their clients/patients were willing to participate (n = 8), technology was intuitive to use (n = 6), their clients/patients felt safe using VR technology (n = 6), clients/patients had positive feedback regarding content (n = 6), and VR placed no strain on their client/patient-provider relationship (n = 6).

### 3.4 Training

Regarding training about the application of VR content in psychiatric/medical treatment (i.e. how to use the virtual spaces in a clinical setting to provide treatment), 52.9% (n = 9) of respondents reported having received training. The greatest percentage of these providers worked directly with or as researchers to create the training (n = 4, 44.4%). Other providers reported having performed self-directed research (n = 3, 33.3%), received clinical supervision (n = 1, 11.1%), or taken a course (n = 1, 11.1%). Length of formal training included less than half a day (n = 1, 16.7%), half to a full day (n = 1, 16.7%), and more than a full day (n = 4, 66.7%). Of those who received training regarding VR content, the most common VR applications used included Psious (n = 3) and Virtually Better (n = 2); by contrast, of those providers who reported not having received training on VR content, the most common application was KindVR (n = 2). All the providers who received training on the VR content found the training to be beneficial. Of those 47.1% (n = 8) who did not receive training on the VR content, six providers (n = 6, 75%) reported that training would have been beneficial.

With regards to training in use of VR as a therapeutic tool, 88.2% (n = 15) of providers reported having received training about how to use their VR platform (i.e. how to navigate the application), with the majority of providers receiving training in person (n = 9, 60%). Other providers reported having received online live training (n = 2, 13.3%), completed self-directed research (n = 2, 13.3%), or received written (n = 1, 6.7%) or pre-recorded (n = 1, 6.7%) training to be completed individually. Length of formal training ranged from less than half a day (n = 6, 46.2%), half to a full day (n = 1, 7.7%) and longer than a full day (n = 6, 46.2%). All providers who reported having received training reported having found the training to be beneficial. Of the two providers (n = 2, 11.8%) who did not receive training, one provider felt training would have been beneficial.

Prior to using VR in their practice, 58.8% (n = 10) of providers reported prior experience with VR while 41.2% (n = 7) did not. Upon starting to use VR in their practice, 47.1% (n = 8) of respondents reported feeling completely comfortable with the technology, and 17.7% (n = 3) reported feeling slightly comfortable. However, 11.8% (n = 2) reported feeling slightly uncomfortable and 23.5% (n = 4) reported that they did not feel at all comfortable. Comparing the trained and untrained groups via two proportion z-tests, there was no significant difference in comfort level upon starting to use VR in practice (p = 0.5058), the value that providers believed VR brought to their practice (p = 0.551), or whether providers would recommend VR to others (p = 0.551), though sample groups were fairly small.

### 3.5 Billing

Regarding reimbursement for VR, only 17.6% (n = 3) of providers report billing their patient’s insurance. Of those who bill their patient’s insurance, only one provider reported being able to bill at an enhanced rate (i.e. a higher rate for sessions that include VR compared to similar sessions without VR). Of those who do not bill their patient’s insurance, the majority (n = 10, 71.4%) of providers reported that VR is not billable in their practice. Other providers (n = 4, 28.6%) were unsure of billing, including one provider who does not bill insurance directly and another whose clinical VR use is part of a research study.

## 4 Discussion

The majority of survey respondents practice outpatient medicine in an academic institution and use VR for the treatment of acute pain and/or anxiety related to medical procedures. The results obtained from this study align with previous research, showing that VR is well-received by patients and providers alike. The majority of providers see VR as a valuable tool for their practice that allows for increased patient engagement, treatment individualization, and patient progress towards treatment goal. The slight majority of providers did not find transition to VR use difficult. Those who did commonly cited cost as a barrier, which may be attributed to providers not being able to bill for this service. Most providers received training on their platform which they found beneficial. These findings may augment standardization of VR as a treatment tool in psychiatry and pain management.

### 4.1 Key findings

The first main section of the questionnaire, ‘VR Use Statistics,’ demonstrates that most survey respondents work in an academic institution with outpatient populations. The majority have been using VR for over 3 years in their clinical practice and spend less than 10% of their clinical time using VR.

The second main section of the questionnaire is ‘VR Value in Practice.’ This section highlights that while VR is a newly emerging tool in the medical field, providers currently using VR technology find it beneficial to their clinical practice. The majority of providers surveyed believe that VR helps their clients/patients progress in treatment compared to similar therapy without VR. These subjective findings corroborate previous studies that have shown the effectiveness of VR in treatment of phobias [4, 11, 12, 26–28], anxiety [5, 6, 10, 13, 20, 29], PTSD [2, 7, 14], addictions [8, 30, 31], and pain management [2, 3, 15, 16, 18, 32].

Other studies [22, 25, 33–35] have documented that providers have concerns inherent to both the VR technology itself as well related to their patients’ response to VR use. Several studies found that clinicians were hesitant to use VR due to foreseen issues related to technology (cybersickness, discomfort of headset, etc.), content (unrealistic scenes, grainy display, etc.), and patient perceptions of VR [1, 35, 36]. The present study demonstrates many of these patient-centered concerns to be unfounded. A minority of providers received negative feedback from patients concerned about technology or content. No providers received negative feedback from patients related to poor perceptions of VR as a treatment tool. Additionally, other studies found some clinicians were hesitant to adopt VR due to fear surrounding detrimental effects on their therapeutic relationships with clients/patients [25, 33, 36]. However, the current study shows that 58% of providers find that in reality, VR allows them to have closer relationships with clients/patients. Furthermore, only 12% of providers felt rushed when using VR with clients/patients. This discordance between hypothetical concerns and actual experience may suggest anxiety related to VR use stems from unfamiliarity with new technology and hesitancy to change current clinical practices rather than intrinsic problems with VR use itself.

The third main section of the questionnaire is titled ‘Onboarding VR in Practice’. Despite previous studies which emphasize non-VR-using providers’ perceptions of difficulty surrounding VR implementation [19, 22, 24, 25, 33], this study finds that the majority of the providers did not find the transition to VR use challenging. Of the minority who did find the transition to be a challenge, initial cost was the most cited issue. This finding aligns with current research demonstrating cost as the most commonly referenced barrier to VR implementation [19, 25, 33]. Additional obstacles to VR clinical implementation cited by providers include the associated learning curve and sufficiently staffing the clinic. In relation to training and onboarding, previous research found that many providers had concerns related to lack of training and potential technical difficulties [19, 24, 25, 33]. The present study demonstrates that the majority of providers received training on the VR platform, and approximately half of the providers received training on the VR content. Those providers found training to be beneficial. Additionally, the majority of providers found VR applications to be intuitive to use for both themselves and their clients/patients. The discrepancy between these positive findings and the previous research highlighting concerns related to the technical aspects of VR suggest that the quality and frequency of training has likely improved. These results are encouraging, as more robustly developed training may eliminate many providers’ technical concerns.

The fourth main section of the questionnaire, ‘Billing/reimbursement of VR in practice,’ shows that the majority of providers are unable to bill for their VR use. The barrier of cost stated above may be eliminated once VR is accepted as a billable service, and thus cost can be attenuated.

The fifth main section of the questionnaire, ‘Clinical Use of VR in Practice,’ found that the majority of providers report using VR to treat more than one patient population, most commonly those experiencing acute pain or anxiety during medical procedures. Some participants reported that they were not currently using VR, but in the future would like to use VR, with various other client/patient populations including chronic pain secondary to an underlying medical condition and acute pain/anxiety during medical procedures.

### 4.2 Limitations

Given that the authors created the VR survey for the purposes of this study, reliability and validity could not be determined. Due to the limited time frame under which the study was conducted as well as the small cohort of providers currently using VR in clinical practice, a small number of surveys were collected. Only 22 surveys were obtained; thus, the sample size may not be indicative of the population as a whole. With such a low response rate, the possibility of sampling bias must be considered. Providers were selected via online presence on VR application websites, news articles and research papers. These providers, especially those who chose to be included on application websites, more likely had a positive experience using VR. Using only this population may have introduced sampling bias, increasing the positive responses compared to the general population of providers using VR with their patients. Additionally, the subset of academic providers identified through involvement in research may collectively have a different view of therapeutic use of VR than the general population of providers.

Finally, contacted providers were asked to forward the survey to any colleagues using VR in their clinical practice. It is unknown how many responses were generated through this method as survey responses were anonymous. However, it is possible that a portion of the 22 responses may not have been from the original selected pool of VR users, but instead referrals.

### 4.3 Future directions

While this small survey suggests that providers overwhelmingly perceive benefit from VR usage in their clinical practice, there remain perceived and real obstacles to the implementation of VR. A larger, randomized controlled trial (RCT) of VR usage in clinical practice could be beneficial in determining and addressing barriers to VR usage. This qualitative study is a snapshot of user experience of VR technology in therapy, and we suggest future research examining VR use over time such as the aforementioned RCT. In addition, focus groups comprising providers both currently using and not using VR clinically may further elucidate reasons for limited VR clinical implementation and allow providers using VR to share positive experiences with their peers. Petitioning insurance companies to allow billing for VR, including at enhanced rates of service, will also be key in ensuring optimal adoption of this technology.

Future research should also focus on identifying clinical subpopulations for which VR may be particularly effective. Most providers in the present study reported only using VR with a minority of their patient population. From this observation arises the question of whether there exists certain patient or illness characteristics that predict a stronger or weaker response to VR treatment. Outcomes from these studies could provide better direction for providers interested in integrating VR into their clinical practice. Given that training was identified as beneficial to all providers, research should further explore the types of training offered to providers and their effectiveness.

Additionally, future work should focus on further development of VR usage in telemedicine. As one provider commented, VR was extremely beneficial during the COVID-19 pandemic when in-person visits were limited. To date, little research has been directed towards this usage of VR delivered remotely or via telehealth. As virtual healthcare visits become more common, this application of VR could prove extremely valuable.

## 5 Final conclusions

The current study demonstrates that VR as a treatment adjunct is generally well-received by patients and providers, allowing increased patient engagement and treatment individualization. These findings corroborate previous research. While many perceived barriers to implementing VR do not hold true in practice, associated costs are commonly identified as an impediment. Offering VR training for providers also mitigates many of their technical concerns. These findings are clinically relevant because the experiences of providers currently using VR will guide new providers through obstacles encountered during the onboarding process. Disseminating this knowledge among colleagues will propel VR forward as a standardized treatment in psychiatric therapy.

## Supporting information

S1 File Full provider survey. (DOCX)

S1 Data (CSV)

## References

[1] Garrett BM, Tao G, Taverner T, Cordingley E, Sun C. Patients perceptions of virtual reality therapy in the management of chronic cancer pain. 2020 May 12;6(5):e03916. doi: 10.1016/j.heliyon.2020.e03916 32426540 PMC7226660

[2] Nusser M, Knapp S, Kramer M, Krischak G. Effects of virtual reality-based neck-specific sensorimotor training in patients with chronic neck pain: A randomized controlled pilot trial. 2021 Feb 10;53(2):jrm00151–2786. doi: 10.2340/16501977-2786 33369684 PMC8814879

[3] Smith V, Warty RR, Sursas JA, Payne O, Nair A, Krishnan S, et al. The Effectiveness of Virtual Reality in Managing Acute Pain and Anxiety for Medical Inpatients: Systematic Review. 2020 Nov 2;22(11):e17980. doi: 10.2196/17980 33136055 PMC7669439

[4] Botella C, Fernandez-Alvarez J, Guillen V, Garcia-Palacios A, Banos R. Recent Progress in Virtual Reality Exposure Therapy for Phobias: A Systematic Review. 2017 Jul 1;19(7):42-017-0788–4. doi: 10.1007/s11920-017-0788-4 28540594

[5] Maples-Keller JL, Bunnell BE, Kim SJ, Rothbaum BO. The Use of Virtual Reality Technology in the Treatment of Anxiety and Other Psychiatric Disorders. 2017 Jun 1;25(3):103–13. doi: 10.1097/HRP.0000000000000138 28475502 PMC5421394

[6] Carl E, Stein AT, Levihn-Coon A, Pogue JR, Rothbaum B, Emmelkamp P, et al. Virtual reality exposure therapy for anxiety and related disorders: A meta-analysis of randomized controlled trials. 2019 Jan 1;61:27–36. doi: 10.1016/j.janxdis.2018.08.003 30287083

[7] Kothgassner OD, Goreis A, Kafka JX, Van Eickels RL, Plener PL, Felnhofer A. Virtual reality exposure therapy for posttraumatic stress disorder (PTSD): a meta-analysis. 2019 Aug 19;10(1):1654782. doi: 10.1080/20008198.2019.1654782 31489138 PMC6713125

[8] Emmelkamp PMG, Meyerbroker K. Virtual Reality Therapy in Mental Health. 2021 May 7;17:495–519. doi: 10.1146/annurev-clinpsy-081219-115923 33606946

[9] Chuan A, Zhou JJ, Hou RM, Stevens CJ, Bogdanovych A. Virtual reality for acute and chronic pain management in adult patients: a narrative review. 2021 May 1;76(5):695–704. doi: 10.1111/anae.15202 32720308

[10] Benbow AA, Anderson PL. A meta-analytic examination of attrition in virtual reality exposure therapy for anxiety disorders. 2019 Jan 1;61:18–26. doi: 10.1016/j.janxdis.2018.06.006 30646997

[11] Wechsler TF, Kumpers F, Muhlberger A. Inferiority or Even Superiority of Virtual Reality Exposure Therapy in Phobias?-A Systematic Review and Quantitative Meta-Analysis on Randomized Controlled Trials Specifically Comparing the Efficacy of Virtual Reality Exposure to Gold Standard in vivo Exposure in Agoraphobia, Specific Phobia, and Social Phobia. 2019 Sep 10;10:1758. doi: 10.3389/fpsyg.2019.01758 31551840 PMC6746888

[12] Salehi E, Mehrabi M, Fatehi F, Salehi A. Virtual Reality Therapy for Social Phobia: A Scoping Review. 2020 Jun 16;270:713–7. doi: 10.3233/SHTI200253 32570476

[13] Maples-Keller JL, Yasinski C, Manjin N, Rothbaum BO. Virtual Reality-Enhanced Extinction of Phobias and Post-Traumatic Stress. 2017 Jul 1;14(3):554–63. doi: 10.1007/s13311-017-0534-y 28512692 PMC5509629

[14] McLay RN, Graap K, Spira J, Perlman K, Johnston S, Rothbaum BO, et al. Development and testing of virtual reality exposure therapy for post-traumatic stress disorder in active duty service members who served in Iraq and Afghanistan. 2012 Jun 1;177(6):635–42. doi: 10.7205/milmed-d-11-00221 22730837

[15] Guo C, Deng H, Yang J. Effect of virtual reality distraction on pain among patients with hand injury undergoing dressing change. 2015 Jan 1;24(1–2):115–20. doi: 10.1111/jocn.12626 24899241

[16] Hoffman HG, Chambers GT, Meyer WJ, Arceneaux LL, Russell WJ, Seibel EJ, et al. Virtual reality as an adjunctive non-pharmacologic analgesic for acute burn pain during medical procedures. 2011 Apr 1;41(2):183–91. doi: 10.1007/s12160-010-9248-7 21264690 PMC4465767

[17] Kipping B, Rodger S, Miller K, Kimble RM. Virtual reality for acute pain reduction in adolescents undergoing burn wound care: a prospective randomized controlled trial. 2012 Aug 1;38(5):650–7. doi: 10.1016/j.burns.2011.11.010 22348801

[18] JahaniShoorab N, Ebrahimzadeh Zagami S, Nahvi A, Mazluom SR, Golmakani N, Talebi M, et al. The Effect of Virtual Reality on Pain in Primiparity Women during Episiotomy Repair: A Randomize Clinical Trial. 2015;40(3):219–24. 25999621 PMC4430883

[19] Segal R, Bhatia M, Drapeau M. Therapists’ perception of benefits and costs of using virtual reality treatments. 2011 Feb 1;14(1–2):29–34. doi: 10.1089/cyber.2009.0398 21329440

[20] Badke CM, Essner BS, O’Connell M, Malakooti MR. An Innovative Virtual Reality Experience in the PICU: A Pilot Study. 2019 Jun 1;20(6):e283–6. doi: 10.1097/PCC.0000000000001917 30920437

[21] Dascal J, Reid M, IsHak WW, Spiegel B, Recacho J, Rosen B, et al. Virtual Reality and Medical Inpatients: A Systematic Review of Randomized, Controlled Trials. 2017 Feb 1;14(1–2):14–21. 28386517 PMC5373791

[22] Boeldt D, McMahon E, McFaul M, Greenleaf W. Using Virtual Reality Exposure Therapy to Enhance Treatment of Anxiety Disorders: Identifying Areas of Clinical Adoption and Potential Obstacles. 2019 Oct 25;10:773. doi: 10.3389/fpsyt.2019.00773 31708821 PMC6823515

[23] Bisso E, Signorelli MS, Milazzo M, Maglia M, Polosa R, Aguglia E, et al. Immersive Virtual Reality Applications in Schizophrenia Spectrum Therapy: A Systematic Review. 2020 Aug 22;17(17): doi: 10.3390/ijerph17176111 32842579 PMC7504018

[24] Schwartzman D, Segal R, Drapeau M. Perceptions of virtual reality among therapists who do not apply this technology in clinical practice. 2012 Aug 1;9(3):310–5. 10.1037/a0026801 22867123

[25] Kramer TL, Pyne JM, Kimbrell TA, Savary PE, Smith JL, Jegley SM. Clinician perceptions of virtual reality to assess and treat returning veterans. 2010 Nov 1;61(11):1153–6. doi: 10.1176/ps.2010.61.11.1153 21041358

[26] Costa RTD, Carvalho MR, Ribeiro P, Nardi AE. Virtual reality exposure therapy for fear of driving: analysis of clinical characteristics, physiological response, and sense of presence. 2018 Feb 15;40(2):192–9. doi: 10.1590/1516-4446-2017-2270 29451586 PMC6900765

[27] Minns S, Levihn-Coon A, Carl E, Smits JAJ, Miller W, Howard D, et al. Immersive 3D exposure-based treatment for spider fear: A randomized controlled trial. 2018 Aug 1;58:1–7. doi: 10.1016/j.janxdis.2018.05.006 29909286

[28] Donker T, Cornelisz I, van Klaveren C, van Straten A, Carlbring P, Cuijpers P, et al. Effectiveness of Self-guided App-Based Virtual Reality Cognitive Behavior Therapy for Acrophobia: A Randomized Clinical Trial. 2019 Jul 1;76(7):682–90. doi: 10.1001/jamapsychiatry.2019.0219 30892564 PMC6583672

[29] Shamri Zeevi L. Making Art Therapy Virtual: Integrating Virtual Reality Into Art Therapy With Adolescents. 2021 Feb 4;12:584943. doi: 10.3389/fpsyg.2021.584943 33613377 PMC7889518

[30] Bordnick PS, Copp HL, Traylor A, Graap KM, Carter BL, Walton A, et al. Reactivity to cannabis cues in virtual reality environments. 2009 Jun 1;41(2):105–12. doi: 10.1080/02791072.2009.10399903 19705672 PMC4104948

[31] Son JH, Lee SH, Seok JW, Kee BS, Lee HW, Kim HJ, et al. Virtual Reality Therapy for the Treatment of Alcohol Dependence: A Preliminary Investigation With Positron Emission Tomography/Computerized Tomography. 2015 Jul 1;76(4):620–7. doi: 10.15288/jsad.2015.76.620 26098039

[32] Wang E, Thomas JJ, Rodriguez ST, Kennedy KM, Caruso TJ. Virtual reality for pediatric periprocedural care. 2021 Jun 1;34(3):284–91. doi: 10.1097/ACO.0000000000000983 33935176

[33] Lindner P, Miloff A, Zetterlund E, Reuterskiöld L, Andersson G, Carlbring P. Attitudes toward and familiarity with virtual reality therapy among practicing cognitive behavior therapists: a cross-sectional survey study in the era of consumer VR platforms. Frontiers in psychology. 2019 Feb 8;10:176. doi: 10.3389/fpsyg.2019.00176 30800086 PMC6376952

[34] Guillen V, Banos RM, Botella C. Users’ Opinion About a Virtual Reality System as an Adjunct to Psychological Treatment for Stress-Related Disorders: A Quantitative and Qualitative Mixed-Methods Study. 2018 Jun 22;9:1038. doi: 10.3389/fpsyg.2018.01038 29988491 PMC6024567

[35] Kramer TL, Savary PE, Pyne JM, Kimbrell TA, Jegley SM. Veteran perceptions of virtual reality to assess and treat posttraumatic stress disorder. 2013 Apr 1;16(4):293–301. doi: 10.1089/cyber.2013.1504 23574368 PMC3624696

[36] Gunter RW, Whittal ML. Dissemination of cognitive-behavioral treatments for anxiety disorders: Overcoming barriers and improving patient access. Clinical psychology review. 2010 Mar 1;30(2): 194–202. doi: 10.1016/j.cpr.2009.11.001 19942331

