# Supplementary material for: Provider experiences of virtual reality in clinical treatment
Source: PLoS One. 2021 Oct 29;16(10):e0259364. doi: 10.1371/journal.pone.0259364 (PMC8555834; doi:10.1371/journal.pone.0259364)
Supplement: S1 File — (DOCX) [file pone.0259364.s001.docx]

**Full Provider Survey**

We are conducting research on the use of virtual reality (VR) as a therapeutic tool. This research is being conducted within Thomas Jefferson University. The Thomas Jefferson University IRB has approved this study as exempt (#20E.634).

In this questionnaire, you are reporting on the use of VR as a tool to provide therapy for your clients/patients. We will ask you about the value VR has for your practice, the process of implementation, and technicalities of use.

Participation in this study is voluntary and you may discontinue your participation at any time. If you do not feel comfortable answering a question, please feel free to skip it. All responses are anonymous. Any information provided may be used to better understand the implementation and use of VR as a therapeutic tool. Your anonymous responses may be used in future publications to improve collective understanding of VR as a treatment tool.

Thank you for your consideration.

Christine Vincent

Margaret Eberts

C. Virginia O’Hayer

Do you wish to continue? ☐yes ☐no

**VR Use Stats**

*The following section of questions are related to the general use of VR in your clinical setting.*

1. What is your primary clinical setting?

☐Community practice (individual)

☐Community practice (group)

☐Academic Institution

☐Other __________

1. How would you describe your primary clinical setting?

☐ Outpatient

☐ Inpatient

☐ Mix of outpatient and inpatient

1. What VR platform(s) do you use in your practice? _____________
2. What VR application(s) do you use in your practice? _______
3. For how long have you been using VR in your practice?

☐Less than 6 months

☐6 months to less than one year

☐1-3 years

☐ Over 3 years

1. What percentage of your clinical time is spent using VR technology with clients/patients?

☐0-10%

☐11-25%

☐26-50%

☐51-75%

☐76-100%

**VR Value in Practice:**

*The next section of questions relate to the effect you perceive VR has on your practice. Please indicate your experience related to the following statements.*

|  | Strongly Agree | Mildly Agree | Mildly Disagree | Strongly Disagree |
| --- | --- | --- | --- | --- |
| VR treatment has been a valuable tool in my practice. |  |  |  |  |
| VR allows me to more accurately diagnose clients/patients. |  |  |  |  |
| VR allows me to individualize treatment to various clients/patients. |  |  |  |  |
| My clients/patients seem more engaged in their VR treatment than in traditional methods. |  |  |  |  |
| VR has helped my clients/patients with their progress in treatment compared with similar methods without VR. |  |  |  |  |
| I have received positive feedback from my clients/patients regarding the use of VR. |  |  |  |  |
| I have received negative feedback from my clients/patients regarding VR and they have attributed this to a problem with content (unrealistic scenes, grainy display, etc). |  |  |  |  |
| I have received negative feedback from my clients/patients regarding VR and they have attributed this to a problem with technology (cybersickness, applications are not intuitive to use, discomfort of equipment, etc). |  |  |  |  |
| I have received negative feedback from my clients/patients regarding VR and they have a problem with the general perception of VR as a treatment tool. |  |  |  |  |
| Using VR has allowed me to have closer relationships to my clients/patients. |  |  |  |  |
| When using VR, I spend more time with clients/patients than I would using other treatment techniques. |  |  |  |  |
| I often felt rushed when using VR with my clients/patients. |  |  |  |  |
| Using VR in my practice presents more technical issues than I would otherwise experience. |  |  |  |  |
| VR apps for therapeutic use are generally intuitive for me to use. |  |  |  |  |
| VR apps for therapeutic use are generally intuitive for clients/patients to use. |  |  |  |  |
| I would recommend the use of VR as a treatment  tool to other providers in my field. |  |  |  |  |

Please comment on any other significant effects VR has on your practice: _______________________

**Onboarding VR in Practice**

*The next set of questions relates to the process in which you decided to engage in the use of VR.*

1. How did you hear about VR as an option in your practice?

☐ Colleague in your practice

☐ Colleague in a different practice

☐ Website

☐ Medical conference

☐ In your training program

☐ Other: (please specify)

1. Are your colleagues open to using VR as an option for therapy?

☐Yes

☐No

☐I did not discuss VR as a therapeutic tool with other colleagues.

If NO (to #2):

1. You stated that your colleagues are not open to using VR therapeutically. Why not? (Check all that apply)

☐ Too much time/effort is required to learn to use VR.

☐ VR hasn’t been proven to be beneficial therapeutically.

☐ The initial cost of VR is too high.

☐ VR use will complicate billing/insurance procedures.

☐ Fear that clients/patients would not like VR form of therapy.

☐ No specific objections, but my colleagues were content with their current therapeutic options

☐ Other: ___

1. Did you find it a challenge to transition to using VR in your practice?

☐ Yes

☐ No

IF NO (to #3):

1. Why was it not a challenge to transition to using VR in your practice? (check all that apply)

☐ Initial cost was not a problem

☐ Technology was intuitive to use

☐ No training was required

☐ Provided training was sufficient

☐ Ongoing technical support is sufficient

☐ My clients/patients were willing to participate

☐ My clients/patients felt safe using VR technology.

☐ My clients/patients had positive feedback regarding content.

☐ VR placed no strain on my client/patient-provider relationship.

☐ VR seemed to enhance my client’s/patient’s progress in treatment

☐ Other:

IF YES (to #3)

- 1. What were the challenges you identified in your own experience of transition to using VR in your practice? (check all that apply)

☐ Initial cost was a problem

☐ Technology was not intuitive to use

☐ Training was required

☐ Provided training was not sufficient.

☐ Ongoing technical support is not sufficient

☐ My clients/patients were reluctant to participate

☐ My clients/patients felt unsafe using VR technology

☐ My clients/patients had negative feedback regarding content

☐ VR placed a strain on my client/patient-provider relationship

☐ Uncertainty of whether VR was benefitting my clients/patients

☐ Other:

- 1. For each challenge you listed, how did you address it (optional)? _________

*The next set of questions pertain to any training you may have received in using VR. The first group relates to training on your VR platform (for example, navigating the application). The second group relates to training focused on using VR content in a clinical setting (for example, clinical supervision).*

1. Did you have any experience using VR prior to its integration into your practice?

☐ Yes

☐ No

1. What was your comfort level with VR upon beginning to use it in your practice?

not at all comfortable/slightly uncomfortable/slightly comfortable/completely comfortable

1. Did you receive training (formal or self directed) on how to use your VR platform?

☐ Yes

☐ No

If YES (to #6):

1. What type of training on how to use your VR platform did you receive?

☐ In person

☐ Online live

☐ Online pre-recorded

☐ Manual/written training provided by the platform to read on your own

☐ Self directed research (youtube, google search, etc)

☐ Other: ______

1. What was the total time of training on how to use your VR platform you received?

☐ Less than half a day

☐ Half a day to a full day

☐ Longer than one day

☐ My training was self directed

☐ I don’t remember

1. Do you feel this training on how to use your VR platform was beneficial?

☐ Yes

☐ No

If NO (to c):

Why do you feel your training on how to use your VR platform was not beneficial? (open-ended)

IF NO (to #6):

1. Do you think formal training on how to use your VR platform would have been beneficial?

☐ Yes

☐ No

If YES:

What do you believe formal training on how to use your VR platform would provide that you did not have otherwise? (open-ended)

1. Did you receive training (formal or self directed) in using VR content in clinical practice?

☐ Yes

☐ No

IF YES (to #7):

1. What type of training in using VR content in clinical practice did you receive?

☐ Clinical supervision

☐ Paid training

☐ Took a course

☐ Self directed research (Read a book, Journal article, internet search)

☐ Other: ______

1. What was the total time of training in using VR content in clinical practice you received?

☐ Less than half a day

☐ Half a day to a full day

☐ Longer than one day

☐ My training was self directed

☐ I don’t remember

1. Do you feel this training in using VR content in clinical practice was beneficial?

☐ Yes

☐ No

If NO (to c):

Why do you feel your training in using VR as a therapeutic tool in clinical practice was not beneficial? (open-ended)

IF NO (to #7):

1. Do you think formal training in using VR content in clinical practice would have been beneficial?

☐ Yes

☐ No

If YES:

What do you believe training in using VR content in clinical practice would provide that you did not have otherwise? (open-ended)

**Billing/Reimbursement of VR in Your Practice**

*The next section of questions asks details regarding your current use of VR, specifically the process of billing and reimbursement.*

1. When you provide VR, do you bill the client’s/patient’s insurance for the session?

☐ Yes

☐ No

If YES:

1. Are you able to use an enhanced billing rate for VR (ie: are you able to bill a higher rate for sessions that include VR compared to similar sessions without VR)?

☐ Yes

☐ No

☐ I don’t know

If NO:

1. Is VR billable in your practice?

☐ Yes, but I have chosen not to bill for it.

☐ No, VR is not billable in my practice.

☐ Other: (please specify)

**Clinical Use of VR in Your Practice**

*The next section of questions asks details regarding your current use of VR, specifically the conditions in which you use VR as a treatment.*

1. I use VR with clients/patients with the following conditions (check all that apply):

☐ Specific phobia

☐ Social phobia

☐ Generalized anxiety disorder

☐ PTSD

☐ Panic disorder

☐ Pain disorder

☐ Chronic pain secondary to underlying medical condition

☐ Acute pain/anxiety during a medical procedure

☐ Substance use disorders

☐ Other: ___

1. I am not currently, but would like to use VR with the following conditions (check all that apply):

☐ Specific phobia

☐ Social phobia

☐ Generalized anxiety disorder

☐ PTSD

☐ Panic disorder

☐ Pain disorder

☐ Chronic pain secondary to underlying medical condition

☐ Acute pain/anxiety during a medical procedure

☐ Substance use disorders

☐ Other: ___

1. In which of the following locations do your clients/patients engage in VR therapy? (check all that apply)

☐ In person (inpatient)

☐ In person (outpatient)

☐ Client/patient independent use (Client/patient uses app on their own)

☐ Virtually (telehealth)

1. (based on answers to #1)

If SPECIFIC PHOBIA selected:

1. What percentage of your total *specific phobia* client/patient population receives VR treatment?

☐ 0-10%

☐ 11-25%

☐ 26-50%

☐ 51-75%

☐ 76-100%

If SOCIAL PHOBIA selected:

1. What percentage of your total *social phobia* client/patient population receives VR treatment?

☐ 0-10%

☐ 11-25%

☐ 26-50%

☐ 51-75%

☐ 76-100%

If GENERALIZED ANXIETY DISORDER selected:

1. What percentage of your total *generalized anxiety disorder* client/patient population receives VR treatment?

☐ 0-10%

☐ 11-25%

☐ 26-50%

☐ 51-75%

☐ 76-100%

If PTSD selected:

1. What percentage of your total *PTSD* client/patient population receives VR treatment?

☐ 0-10%

☐ 11-25%

☐ 26-50%

☐ 51-75%

☐ 76-100%

If PANIC DISORDER selected:

1. What percentage of your total *panic disorder* client/patient population receives VR treatment?

☐ 0-10%

☐ 11-25%

☐ 26-50%

☐ 51-75%

☐ 76-100%

If PAIN DISORDER selected:

1. What percentage of your total *pain disorder* client/patient population receives VR treatment?

☐ 0-10%

☐ 11-25%

☐ 26-50%

☐ 51-75%

☐ 76-100%

If CHRONIC PAIN SECONDARY TO UNDERLYING MEDICAL CONDITION selected:

1. What percentage of your total *chronic pain secondary to underlying medical condition* client/patient population receives VR treatment?

☐ 0-10%

☐ 11-25%

☐ 26-50%

☐ 51-75%

☐ 76-100%

If ACUTE PAIN/ANXIETY DURING MEDICAL PROCEDURE selected:

1. What percentage of your total *acute pain/anxiety during medical procedure* client/patient population receives VR treatment?

☐ 0-10%

☐ 11-25%

☐ 26-50%

☐ 51-75%

☐ 76-100%

If SUBSTANCE USE DISORDER selected:

1. What percentage of your total *substance use disorder* client/patient population receives VR treatment?

☐ 0-10%

☐ 11-25%

☐ 26-50%

☐ 51-75%

☐ 76-100%

If OTHER: _____ selected:

1. What percentage of your total *other _____* client/patient population receives VR treatment?

☐ 0-10%

☐ 11-25%

☐ 26-50%

☐ 51-75%

☐ 76-100%
